# Profiling the Skeletal Muscle Proteome in Patients on Atypical Antipsychotics and Mood Stabilizers

**DOI:** 10.3390/brainsci12020259

**Published:** 2022-02-12

**Authors:** Kyle J. Burghardt, Griffin Calme, Michael Caruso, Bradley H. Howlett, Elani Sanders, Zaher Msallaty, Abdullah Mallisho, Berhane Seyoum, Yue A. Qi, Xiangmin Zhang, Zhengping Yi

**Affiliations:** 1 Department of Pharmacy Practice, University Eugene Applebaum College of Pharmacy and Health Sciences, Wayne State University, 259 Mack Avenue, Suite 2190, Detroit, MI 48201, USA; griffincalme@wayne.edu (G.C.); gl7527@wayne.edu (B.H.H.); ed6286@wayne.edu (E.S.); 2 Department of Pharmaceutical Science, Eugene Applebaum College of Pharmacy and Health Sciences, Wayne State University, 259 Mack Avenue, Detroit, MI 48201, USA; michael.caruso2@wayne.edu (M.C.); xiangmin.zhang@wayne.edu (X.Z.); Zhengping.yi@wayne.edu (Z.Y.); 3 Division of Endocrinology, School of Medicine, Wayne State University, 4201 St Antoine, Detroit, MI 48201, USA; zmsallaty@wayne.edu (Z.M.); ee5984@wayne.edu (A.M.); bseyoum@wayne.edu (B.S.); 4 Center for Alzheimer’s and Related Dementias, National Institutes of Health, Bethesda, MD 20892, USA; yue.qi@wayne.edu

**Keywords:** skeletal muscle, antipsychotic, mood stabilizer, proteomic

## Abstract

Atypical antipsychotics (AAP) are used in the treatment of severe mental illness. They are associated with several metabolic side effects including insulin resistance. The skeletal muscle is the primary tissue responsible for insulin-stimulated glucose uptake. Dysfunction of protein regulation within the skeletal muscle following treatment with AAPs may play a role in the associated metabolic side effects. The objective of this study was to measure protein abundance in the skeletal muscle of patients on long-term AAP or mood stabilizer treatment. Cross-sectional muscle biopsies were obtained from patients with bipolar disorder and global protein abundance was measured using stable isotope labeling by amino acid (SILAC) combined with high-performance liquid chromatography-electrospray ionization tandem mass spectrometry (HPLC-ESI-MS/MS). Sixteen patients completed muscle biopsies and were included in the proteomic analyses. A total of 40 proteins were significantly different between the AAP group and the mood stabilizer group. In-silico pathway analysis identified significant enrichment in several pathways including glucose metabolism, cell cycle, apoptosis, and folate metabolism. Proteome abundance changes also differed based on protein biological processes and function. In summary, significant differences in proteomic profiles were identified in the skeletal muscle between patients on AAPs and mood stabilizers. Future work is needed to validate these findings in prospectively sampled populations.

## 1. Introduction

The atypical antipsychotics (AAPs) are a medication class whose affinity for dopamine and serotonin receptors are thought to drive their therapeutic efficacy [1]. AAPs are used for the treatment of several mental illnesses including schizophrenia, bipolar disorder, and major depressive disorder. Despite the therapeutic benefits of AAPs, non-compliance with this class of medication is very high and often a result of side effects [2,3]. The most prominent side effects of AAPs are metabolic in nature and include weight gain, insulin resistance and dyslipidemia. Through these side effects, AAPs increase the risk of metabolic syndrome, diabetes, and cardiovascular disease 1.5–3 times compared to the general population [4,5,6,7]. Although the disorders for which AAPs are used carry a drug-naive risk of metabolic dysregulation, recent work has pointed to a potentially direct effect of AAPs on insulin sensitivity [8,9,10]. The exact molecular mechanism of AAP-induced metabolic side effects is not fully known, however several different “omic” areas have been implicated including genetic, epigenetic, lipidomic, and metabolomic [11,12,13,14,15].

A potential limitation of currently reported studies investigating the mechanisms of AAP-induced metabolic side effects is the utilization of peripheral samples such as serum or plasma. These sample sources are clinically accessible and are well-suited for biomarker development, however, due to their heterogeneous cellular makeup, may not reflect the primary tissues that underlie AAP-induced metabolic side effects. Metabolic side effects are likely due to dysregulation in several tissues or organs including the liver, adipose tissue, and skeletal muscle. The skeletal muscle has been demonstrated to be the primary tissue in insulin-stimulated glucose uptake and therefore dysregulation within this tissue is thought to be central to insulin resistance [16,17]. Thus, human skeletal muscle is an ideal candidate tissue for further investigations into the potential effects of AAPs on this tissue’s molecular pathways to help explain peripheral AAP side effects (i.e., metabolic side effects).

Proteomics is a field of study that aims to analyze the protein abundance profile within a given tissue sample or cell. Proteomics utilizes advanced mass spectrometry approaches to characterize the protein abundance of many proteins within a given sample. Protein abundance may be particularly important in dictating protein activity and cellular processes [18]. There have been several studies looking at the effect of mental illness (e.g., schizophrenia) or AAP use on the proteome of clinically obtained peripheral tissues including human serum and plasma and pre-clinical models of the central nervous system [19,20,21,22,23,24,25]. Work in pre-clinical models has identified protein changes in pathways such as lipid homeostasis, mitogen-activated protein kinases or extracellular signal-regulated kinases (MAPK/ERK), and electron transport chain pathways [22,23,26]. Levin and colleagues identified serum global proteomic changes that included several apolipoproteins in the serum of schizophrenia patients compared to controls [24]. Similar changes in apolipoproteins have also been identified in the cerebrospinal fluid and liver of patients with schizophrenia [25]. Jaros and colleagues performed two investigations of serum protein phosphorylation [19,20]. The first study identified concurrent changes in protein abundance and phosphorylation in a set of antipsychotic-naive subjects compared to healthy controls. They identified protein abundance differences in 35 proteins and protein phosphorylation differences in 72 proteins related to acute phase response, lipid and glucose homeostasis (LXR), and retinoic acid signaling (RXR). Although this study did not investigate antipsychotic effects, it provided evidence of proteomic changes at baseline in schizophrenia patients as potential targets for antipsychotic treatment. In a second investigation, Jaros and colleagues identified differences in 45 protein phosphosites in serum after the treatment of patients with olanzapine for six weeks. They identified mixed changes in phosphorylation, of which 26 of the phosphorylated proteins were also identified in their first study. Within this second study, they found that olanzapine treatment may also influence phosphorylation patterns related to acute phase response and LXR and RXR signaling, and that it may “correct” some phosphorylation when compared to healthy controls. Finally, Telford and colleagues investigated the effect of six weeks of olanzapine treatment of serum protein glycosylation [21]. They identified potential effects of olanzapine on digalactosylation and disialylation of serum proteins which may play a role in several critical cellular processes.

Despite investigations into the effect of AAP use on the serum or plasma proteome and the known importance of skeletal muscle proteomic regulation in the pathophysiology of metabolic disease, including insulin resistance, there has only been two targeted proteomics study investigating the effect of an AAP on the protein abundance and regulation in an L6 rat muscle cell model [27,28]. To date, proteomic analyses have not been performed on skeletal muscle of humans treated with AAPs. The aim of this study was to investigate the effect of AAP treatment compared to mood stabilizer treatment on human skeletal muscle proteome as a first step to establishing the potential effects of AAPs on skeletal muscle molecular functions, which can be used in future investigations of AAP-induced metabolic side effects.

## 2. Materials and Methods

### 2.1. Clinical Population and Assessments

All procedures and protocols were approved by the adult medical institutional review board of Wayne State University. Potential participants were recruited through public advertisements and invited to the Wayne State University Clinical Research Services Center (CRSC) to undergo a screening for inclusion into the study following informed consent. Patients were included if they met the following criteria: (1) had a diagnosis of bipolar disorder (I, II, or not otherwise specified) as confirmed by the Mini International Neuropsychiatric Exam conducted by a trained research assistant, (2) on an AAP or mood stabilizer with no dosage changes >25% for 3 or more months, and (3) between the age of 18–65 years. Although antipsychotics have demonstrated mood stabilizing properties, for the purposes of this study, the term “mood stabilizer” is used to categorize patients treated with a guideline medication used for mood stabilization in bipolar disorder other than an antipsychotic [29]. Three months was chosen as a cutoff for dosage changes to capture a patient population on a stable and consistent treatment regimen and to minimize the potential fluid metabolic changes observed early in treatment (e.g., weight gain with antipsychotics, etc.). Participants were excluded for the following: (1) diabetes (2-h oral glucose tolerance test with glucose 200 or greater), (2) primary relative with diabetes, (3) unable to refrain from anticoagulants 7 days before or after biopsy, (4) allergy to lidocaine, or (5) history of bleeding disorder.

Patients were assessed for vitals and anthropometrics, a standard 75-g, 2-h oral glucose tolerance test, blood draws for analysis of glucose and insulin, and a fasting muscle biopsy. Vitals and anthropometrics were measured and included height, weight, blood pressure, heart rate, and body fat by bioelectric impedance (Biodynamics BIA 450 Bioimpedance Analyzer, Biodynamics, Shoreline, WA, USA). Blood draws were performed at baseline and every 30 min during the oral glucose tolerance test. A fasting muscle biopsy was obtained from the *vastus lateralis* using the modified bergstrom needle technique. The muscle tissue was quickly cleaned of blood and connective tissue and immediately frozen in liquid nitrogen [30]. Biopsies were stored at −84 °C until further processing. Glucose was analyzed by a bedside YSI 2300 Stat Plus Glucose Lactate Analyzer (YSI, Yellow Springs, OH, USA). Insulin was analyzed with Alpco Insulin Enzyme-linked immunosorbent assay (ELISA) kits according to manufacturer instructions. The homeostatic model of insulin resistance (HOMA-IR) was used as an index of insulin resistance [31]. Metabolic syndrome was defined according to published criteria [32]. Glucose area-under-the-curve (AUC) and insulin AUC during the OGTT were calculated using the trapezoidal method with Excel (Seattle, WA, USA) as an additional surrogate of insulin resistance [33].

### 2.2. Proteomic Abundance Analysis

Muscle biopsies from 8 participants each from the two groups (AAP and mood stabilizer) were homogenized using a Next Advance Bullet Blender (Model BBY5E) in 8 M urea buffer containing protease inhibitors and phosphatase inhibitors. The lysate was centrifuged, and the supernatant was moved to a new tube and the protein concentration was measured by Bradford protein assay. Three mg of proteins from each biopsy and 300 μg “heavy” Stable Isotope Labeling by Amino Acid (SILAC) labeled protein standards were mixed. The “heavy” SILAC labeled protein standards were from the stock we prepared with SILAC incorporation rate >95%, as described in [34]. The protein mixture underwent reduction (10 mM dithiothreitol (DTT) incubation for 30 min at 55 °C) and alkylation (50 mM iodoacetamide (IAA) incubation for 30 min at room temperature in dark). The buffer for these protein mixtures was exchanged to 40 mM ammonium bicarbonate using an Amicon 5 kD spin column, followed by in-solution trypsin digestion using 60 μg trypsin Protease MS Grade (catalog #PI90058, Fisher scientific, Hampton, NH) for 16 h at 37 °C. After the digestion, the resulting tryptic peptide mixture was passed through an Amicon 10 kD spin column to remove excessive trypsin. The pass throughs containing tryptic peptides were dried by vacuum centrifugation, which removed ammonium bicarbonate. The resulting protein digests were reconstituted with 0.1% TFA in water and analyzed by high-performance liquid chromatography-electrospray ionization tandem mass spectrometry (HPLC-ESI-MS/MS) for protein abundance, according to the method we published earlier [35]. In brief, the peptide mixture was separated with a linear gradient of 5–35% buffer B (0.1% FA in ACN) in 120 min at a flow rate of 300 nL/min on a C_18_-reversed phase column (75 μm ID, 40 cm length) packed in-house with ReproSil-Pur C18-AQ 3 μm resin (Dr. Maisch GmbH) in buffer A (0.1% FA). A nanoflow Dionex Ultimate 3000 UPLC system was online coupled to a Thermo Finnigan LTQ-Orbitrap Lumos fitted with a nanospray flex ion source (Thermo Fisher, San Jose, CA, USA).

A “top 20” data-dependent tandem mass spectrometry approach was utilized to identify peptides in the samples. In a top 20 scan protocol, a full scan spectrum is acquired followed by collision-induced dissociation (CID) mass spectra of the 20 most abundant ions in the survey scan. The survey scan was acquired using the Orbitrap mass analyzer to obtain high mass accuracy and high mass resolution data, and up to 20 of the most intense peptides were selected and subjected to fragmentation in the linear ion trap (LTQ). Dynamic exclusion was set at 30 s. The charge state rejection function was enabled with “unassigned” and “single” charge states rejected. By knowing the accurate mass and fragmentation pattern of the peptide, the peptide’s amino acid sequence can be reliably inferred.

Raw MS files were processed using the MaxQuant software, a popular quantitative proteomics software package [36,37,38]. The database with forward and reversed Uniprot human protein sequences was downloaded from www.uniprot.org (accessed on 9 June 2021) Standard settings in the MaxQuant were applied. Parent mass tolerance was 5 p.p.m., and fragment mass tolerance was 0.5 Da. Two missing trypsin cleavage sites were allowed. Oxidized methionine (M), phosphorylation (STY), and acetylation (protein N-term) were allowed as a variable modification. The false discovery rate (FDR) for both proteins and peptides (with minimum 6 amino acids) was set to 0.01. Only proteins identified with minimum of 2 unique peptides and with FDR for both proteins and peptides ≤0.01 were considered.

To minimize the experimental variation during sample preparation and HPLC-ESI-MS/MS data acquisition, we developed and validated a modified Super-SILAC approach (we now call it Universal-SILAC), in which SILAC labeled protein lysates were spiked-in to each experimental sample and were used as a universal standard for quantification purpose [34,39]. The normalized peak area for non-labeled peptides was calculated by normalizing the peak area of a peptide (PAi) against the sum of the peak area of the isotope-labeled peptide, which were identified in the same sample [34,39]. Please note that traditional SILAC/spike-in Super-SILAC requires both light peptides and corresponding heavy-labeled peptides to be identified for quantification. Unfortunately, identification of only the light or heavy-labeled peptide is a phenomenon commonly observed in traditional SILAC/Super-SILAC experiments, which leads to fewer quantifiable peptides [40]. Our Universal-SILAC approach does not require both light peptides and corresponding heavy-labeled peptides to be identified for quantification, therefore it provides more quantitative information than the traditional SILAC/Super-SILAC quantification. Please see our publication [34,39] for more details. The Universal-SILAC strategy can be easily applied to proteome quantification [34]:

$$Norm:i=\frac{PAi}{Sum\;of\;the\;peak\;area\;for\;the\;labeled\;proteins\;identified\;in\;the\;same\;sample}$$

These normalized intensities were used for statistical analysis of protein abundance differences between groups (i.e., AAP versus mood stabilizer).

### 2.3. Statistical Analysis

Continuous variables are reported as means ± standard error (s.e.) and categorical variables are reported as percentages. Clinical and demographic variables were analyzed between groups by independent student’s t-test, chi-square test, or fisher’s exact test, where appropriate. Fasting insulin, HOMA-IR, and insulin AUC were analyzed by Mann Whitney due to their deviation from normality. A *p* < 0.05 was considered statistically significant for these tests.

In the proteomic analyses, although many proteins were identified, a series of filters were applied to narrow the number of proteins that were used in comparisons between groups to minimize false positives: (1) Identified in ≥4 biopsies out of the 8 AAP or 8 mood stabilizer biopsies; (2) with a fold change greater than 1.5 (i.e., 1.5-fold increase) or less than 0.67 (i.e., 1.5-fold decrease) between the AAP and mood stabilizer biopsies. Independent t-tests were performed to assess effects of AAP and mood stabilizers on protein abundance and *p* < 0.01 was considered statistically significant for this. If a protein was only identified in AAP or a mood stabilizer for at least half of the time (i.e., ≥4 samples), this protein was considered significantly different by default.

### 2.4. Bioinformatics/Pathway Analysis

For an in-silico pathway and bioinformatics analysis, significantly different proteins based on the analysis criteria described above were entered into Ingenuity Pathway Analysis Software (Qiagen, Germantown, MD, USA) by entering the corresponding gene name to the identified protein. The canonical pathway module was performed to analyze significantly enriched pathways based on the entered data. The top-performing pathways that were significant (defined as a false discovery rate (FDR) ≤ 0.1) are presented in the results. The molecule module was used to derive a list of molecules associated with canonical pathways and to present a qualitative analysis of protein location and annotated function that had increased or decreased protein abundance.

## 3. Results

### 3.1. Description of Clinical Population

A total of 16 patients completed the study to be included in the proteomic analysis (Table 1). Fifty percent of the sample were currently treated with AAPs (mean chlorpromazine equivalent dose ± s.e. = 323 ± 44.7) that included quetiapine (*n* = 3), risperidone (*n* = 2), olanzapine (*n* = 2), and asenapine (*n* = 1). The remainder were on mood stabilizers including lamotrigine (*n* = 4; mean dose = 212.5 mg/day), lithium (*n* = 3; mean dose = 650 mg/day), and valproic acid (*n* = 1; 1000 mg/day). Within the AAP group, all were diagnosed with bipolar disorder I except one patient with bipolar disorder II, and within the mood stabilizer group all were diagnosed with bipolar disorder I except two patients with bipolar disorder II. The treatment groups did not significantly differ in any demographic or clinical variable. The AAP group had qualitatively higher levels of fasting and insulin AUC, which is an expected effect of AAP treatment; however, this was not statistically significant [10,41]. The AAP group had one patient meeting metabolic syndrome criteria, while the mood stabilizer group had two. Together, this suggests the groups were comparable on most measured variables except for drug treatment as intended in the study design.

### 3.2. Protein Abundance Analyses

HPLC-ESI-MS/MS analysis indicated that 1110 proteins were identified with a minimum of two unique peptides and with an FDR for both proteins and peptides ≤0.01. As described in the Methods section, a series of filters were used to narrow the number of proteins that were used in comparisons between groups to minimize false positives: (1) Identified in ≥4 biopsies out of the eight AAP or eight mood stabilizer biopsies, and 469 proteins met this criterion; (2) with a fold change greater than 1.5 (i.e., 1.5-fold increase) or less than 0.67 (i.e., 1.5-fold decrease) between the AAP and mood stabilizer biopsies, and 168 proteins met this criterion. Thus, 168 independent t-tests were performed to assess effects of AAP and mood stabilizer on protein abundance. In total, 34 out of the 168 proteins were significantly different (*p* < 0.01) between the AAP and mood stabilizer groups (Table 2).

In addition, if a protein was identified only in one group (AAP or mood stabilizer) and was present in at least half of the samples in that group (≥4 biopsies), we assumed the protein in the other group was too low to be detected, and those proteins were considered to have higher abundance in the detected group by default. Six proteins satisfied this criterion. As a result, in total, the protein abundance for 40 (34 + 6 = 40) proteins were significantly different between AAP and mood stabilizer groups.

### 3.3. Bioinformatics/Pathway Analysis

**Figure 1:**
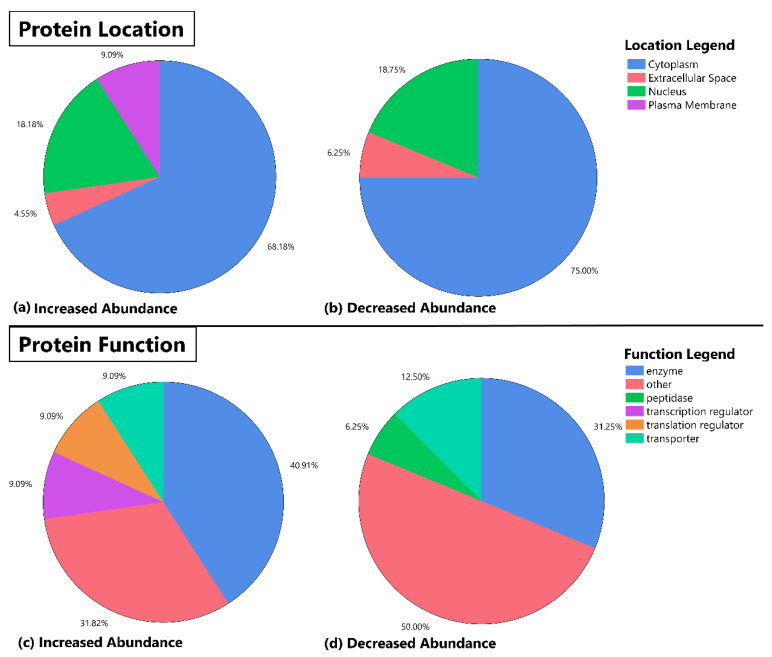
Pie chart representation of protein location and function for identified protein abundance differences between patients on atypical antipsychotics (AAP) and mood stabilizers. This figure depicts the location and function (as determined by the Molecule Module of Ingenuity Pathway Analysis Software) of protein abundancies that were determined to be significantly different in the skeletal muscle of AAP-treated subjects versus mood stabilizer-treated subjects. The top half of the panel depicts protein location (**a**,**b**) and the bottom half of the panel shows the distribution of annotated function for each identified protein (**c**,**d**). The increased abundance and decreased abundance refer to increases or decreases in the AAP group relative to the mood stabilizer group.

Protein function and location for significant proteins identified from our analysis (Table 2) are shown in Figure 1. Overall, most proteins were found in the cytoplasm followed by the nucleus for either increased or decreased proteins in the AAP group. The most common function for the identified proteins was enzyme in the increased abundance and “other” for the decreased abundance. Of note, identified proteins from our abundance analysis saw an enrichment in transcription and translation regulators in the proteins with increased abundance, which was not observed in the proteins with decreased abundance in the AAP group.

In-silico pathway analysis of significantly altered protein abundance sites revealed an enrichment of 107 pathways, with 21 below an FDR *p* of 0.1. The most significant enrichment was in p70S6K signaling and cell cycle pathways. A list of the top pathways is presented in Table 3.

## 4. Discussion

The proteomic abundance analyses described here identified 40 proteins that either increased (22) or decreased (18) in the skeletal muscle of AAP compared to mood stabilizer-treated patients. To our knowledge, this is the first proteomic investigation of the effects of AAPs on the skeletal muscle, a tissue that could potentially play a role in the side effects of AAPs. Most proteomic studies on antipsychotic or mood stabilizer use have been performed in the blood (see introduction for review of such studies) and have identified a wide array of proteins (hundreds) and their subsequent pathways that may be influenced by treatment. When comparing the findings here to those studies, there appears to be distinct candidate pathways that are identified for future investigation, however, many of these proteomic studies had differing study designs (beyond blood-based proteomics) that make comparisons difficult. Future work will need to understand what proteomic signatures in the skeletal muscle are reflected in the clinically accessible blood for potential utility in directing treatment. As we discuss below, some of the main results of this proteomics study are supported by other studies in the literature, suggesting an overlap in findings between blood and skeletal muscle when considering a particular protein or pathway.

### 4.1. Pathway Analysis

The in-silico bioinformatics and pathway analyses of the significantly changed proteins revealed several canonical pathways that were potentially associated with AAP treatment when compared to mood stabilizer treatment. The identified pathways included various signaling pathways, several folate-related pathways, and glucose-related pathways. The top proteomic pathway, p70S6K signaling, is downstream of phosphoinositide-3-kinase (PI3K) and has been demonstrated to be involved in insulin sensitivity, particularly within metabolic tissues such as skeletal muscle and adipose tissue [42,43,44]. Additionally, the canonical pathway analysis also found that four skeletal muscle pathways related to the PI3K and glucose metabolism pathways were influenced by AAP treatment. These pathways included the insulin-like growth factor 1 (IGF-1) signaling (an activator of the protein kinase (AKT) pathway) [45], the PI3K/AKT signaling pathway itself [46], 14-3-3-mediated signaling (binds to proteins found in AKT pathway) [47], and HIPPO pathway signaling (works alongside the 14-3-3 pathway) [48]. As the skeletal muscle is the primary tissue involved in insulin-stimulated glucose uptake, the potential effects on these various glucose metabolism pathways could aid in describing the direct effects of AAPs on insulin sensitivity [10]. As previously described, work in L6 muscle models has demonstrated that olanzapine may cause dysregulation in the PI3K/AKT and glycogen synthesis pathways, and our group has identified epigenetic dysregulation of the AKT pathway in the skeletal muscle [27,28,49]. The pathway analysis results from this study are the first to suggest that there may be protein dysregulation occurring within these related pathways of the skeletal muscle in-vivo. The pathophysiology of insulin resistance and the role of skeletal muscle molecular mechanisms continues to be an active area of research in the field of diabetes and endocrinology. It is not known if the insulin resistance caused by AAPs is facilitated by similar or distinct mechanisms. An in-depth examination of the proteomic abundance, regulation, and interactions of the PI3K/AKT pathway and other glucose metabolism pathways is needed to better understand the potential skeletal muscle mechanisms of AAP-induced insulin resistance.

PI3K/AKT is also involved in telomerase signaling, a primary consideration of the identified “Cell Cycle: G2/M DNA Damage Checkpoint Regulation” pathway, which suggests a role for AAPs on genetic integrity and function. Telomeres, repetitive ribonucleoprotein complexes on the end of chromosomes, are pivotal to chromosome protection and cell division [50]. Compromised telomeres have been linked to many different exposures (e.g., environmental, etc.) and disease states, thus the role of telomeres in the mechanisms of medication efficacy or side effects is potentially large [51,52,53,54,55]. Additionally, several pathways related to cell survival and apoptosis were also identified, which should be considered in the overall view of cellular stability. In support of a potential role of telomere signaling and integrity in psychopharmacologic mechanisms, work has shown effects of both AAPs and mood stabilizers on telomere integrity and treatment outcomes [56,57,58,59]. Considering these past findings and our findings here, further research into telomeres and AAP treatment in a tissue-specific manner may be warranted.

Folate metabolism was also implicated from our pathway analysis with the identification of enrichment in the pathways of (1) diphthamide biosynthesis, (2) tetrahydrofolate salvage from 5,10-methenyltetrahydrofolate, (3) folate polyglutamylation, (4) histidine degradation III, (5) folate transformations I and (6) purine nucleotides de novo biosynthesis II. The dipthamide biosynthesis pathway utilizes s-adenosyl methionine (SAM, a product of the folate pathway) for synthesis of dipthamide and may have a role in translation regulation [60]. The tetrhydrofolate salvage and folate polyglutamylation pathways provide additional folate sources and prepare better folate substrates, respectively, providing necessary fuel for the cycle and its associated reactions. The histidine degradation pathway feeds into both glutamate degradation and folate pathways. The folate transformations I pathway includes the various general folate transformations within the main folate pathway itself, while the purine nucleotides de novo biosynthesis II pathway utilizes 10-formyl-tetrahydrofolate twice to make tetrahydrofolic acid in the production of purines. Together, these pathways involve the activation of folate for use in the body in various reactions and act as a primary source of methyl donors (as seen in the purine pathway). Of note, a line of work has looked at folate metabolism in relation to AAP treatment, including both efficacy and side effects, and found that specific genetic variation of key folate enzyme may be associated with AAP outcomes and further related to changes in gene function [61,62,63,64]. The findings here add to this body of work by being the first to demonstrate associations with these pathways with AAP treatment in human skeletal muscle.

### 4.2. Proteomic Changes Depend on Protein Location and Function

The changes in proteomic profiles differed based on annotated location and function of the protein. Approximately 65–75% of proteins that had either increased or decreased abundance were located within the cytoplasm, suggesting a relatively large effect of medication treatment on the regulation of these proteins. Twenty percent of proteins with either increased or decreased abundance were found in the nucleus, while approximately 5% of each group were found in the endoplasmic reticulum. Increased abundance had ~9% of proteins in the plasma membrane while no proteins were found in this location for decreased abundance.

Protein function identified some key differences between proteins with increased and decreased abundance. For example, proteins with decreased abundance had 6.3% of proteins categorized as peptidases while proteins with increased abundance had none in this category. Similarly, 9.1% of increased proteins were found to be transcription and translation regulators. Future work could utilize these findings to understand the entire range (e.g., systems biology) of molecular associations and effects on AAP treatment response.

### 4.3. Individual Protein Abundance Differences between Treatment Groups

Many individual protein differences were identified in this study, and a few have relevance to AAPs or mood stabilizers. Several proteins showed decreased abundance in patients on AAPs versus mood stabilizers. For example, adenosine triphosphate (ATP) synthase subunit delta (ATP5D) is a subunit of mitochondrial ATP synthase, which plays a key role in ATP synthesis and the oxidative phosphorylation metabolic pathway, and regulating mitochondrial function was lower in the muscle of AAP-treated patients [65]. Given the known role of fatty acids and glucose for oxidative phosphorylation, there have been several investigations into this pathway and the effects of antipsychotics across various tissues [66,67,68]. Similarly, oxidative phosphorylation may play a role in the effects of mood stabilizers as well [69,70,71].

In addition to ATP5D, our data identified other proteins of interest with lower abundance in the AAP group, such as four and a half LIM domains protein 1 (FHL1), fatty acid-binding protein 3 (FABP3), heat shock 70 kDa protein 6/7 (HSPA6/7), and myosin-3 (MYH3). FHL1 and MYH3 have been shown to have decreased RNA levels in skeletal muscle following the infusion of olanzapine in rat models [72]. FABP3 gene expression may also be influenced by antipsychotics, lithium, and valproic acid [73]. Furthermore, HSPA6/7 are chaperone proteins of the heat shock family, which may enhance metabolic profiles in skeletal muscle and serve as a defense system against insulin resistance and T2D [74]. O-(3-piperidino-2-hydroxy-1-propyl)nicotinic amidoxime (BGP-15), an insulin sensitizer drug candidate shown to increase heat shock protein expression, can prevent metabolic side effects of AAP in-vivo [75]. Although this is not an exhaustive discussion of the individual protein changes and their relevance to AAP and mood stabilizer treatment, it demonstrates that the results have both novel and literature-supporting elements.

### 4.4. Limitations

Although this is the first study to examine skeletal muscle proteomic abundance in patients treated with AAPs compared to mood stabilizers, a few limitations should be considered. First, this was a cross-sectional study with a limited sample size, however, proteomic examination of the biopsied skeletal muscle from a clinical population with mental illness has not been previously done and we were still able to identify significant differences. Second, all “omic” type investigations involve multiple statistical tests, raising the issue of potential false positives. This study employed a combination of fold change and lowered *p*-value threshold to account for this. The false discovery rate (FDR) has emerged as a powerful method to correct for multiple statistical comparisons, which offers an excellent balance between false positives and false negatives. FDR is defined as [76,77,78]:

$$\mathrm{FDR}=\frac{\#\;\mathrm{of}\;\mathrm{false}\;\mathrm{positive}\;\mathrm{features}}{\#\;\mathrm{of}\;\mathrm{significant}\;\mathrm{features}}$$

Among the 34 significant proteins (excluding non-detected proteins) in this study with *p* < 0.01, there could be 1.68 false positives (168 ∗ 0.01 = 168). Thus, the FDR is 0.049 (1.68/34 = 0.049) for this study, which is within a generally accepted range for FDR cutoffs. Nonetheless, follow-up studies in additional cohorts of prospectively treated patients should be pursued to validate the findings from this study. Within our study, we measured abundance using an untargeted approach. However, the ability to measure relative quantitative proteomic abundance changes by an unbiased fashion in patients only differing based on pharmacologic treatment is important and offers hypothesis generation for future studies, as discussed above. Individual AAP and mood stabilizer medications were not uniform within each group and this heterogeneity could lead to differences in pharmacokinetic and side effect profiles that must be considered when interpreting the results here. Future work could utilize the findings here and focus skeletal muscle proteomic analyses on the most metabolically adverse AAPs, such as olanzapine, in a prospective format. Our groups were mostly similar in terms of demographics and clinical characteristics. The AAP group did have qualitatively higher values of glucose AUC, insulin AUC, and HOMA-IR compared to the mood stabilizer group, however, these differences were not statistically significant which would have been hypothesized based on the known effects of AAPs on insulin resistance. This lack of difference could be from a small sample size. The HOMA-IR values in both groups are elevated when compared to suggested cutoffs in general population studies, which could be due to a combination of high body weight and treatment with medications known to influence HOMA-IR [79,80,81]. One HOMA-IR value could be considered an outlier in the AAP group (27.9). Although all patients had blood drawn in a fasting condition, this value is greater than two standard deviations from the mean. Nevertheless, the differences were not significant between the groups for HOMA-IR (*p* = 0.5), and with removal of this probable outlier the group difference remains non-significant (*p* = 0.7); the group means remain higher compared to most proposed cutoffs. Some studies with AAPs in obese patients have identified high HOMA-IR values, and HOMA-IR appears correlated to weight [82,83,84]. Nevertheless, this high HOMA-IR value should be a consideration when interpreting the proteomic findings here. A potential benefit could be that this allows us to better determine the skeletal muscle proteomic changes with AAP treatment while reducing the possibility that skeletal muscle insulin resistance itself was the cause of proteomic changes. This provides rationale for future prospective work analyzing proteomic changes before and after AAP treatment.

## 5. Conclusions

This study reported, for the first time, differences in skeletal muscle proteome between patients treated with AAPs versus mood stabilizers. Pathway analysis identified significantly influenced pathways related to glucose metabolism, folate metabolism, and apoptosis. Future work in prospectively sampled populations is needed to validate these findings and begin to identify pathways as possible targets to reduce the morbidity and mortality associated with AAP-induced insulin resistance and other metabolic side effects.

## Figures and Tables

**Table 1:** Description of Clinical Sample Population. Values presented as mean ± s.e. with range (min, max) or percent. Average length on treatment refers to antipsychotic or mood stabilizer. BMI = Body Mass Index; * reflects the number of months that have elapsed since the medication was first prescribed.

	Atypical Antipsychotic (*n* = 8)	Mood Stabilizer (*n* = 8)	*p*-Value
Age (years)	45.5 ± 5.1 (26, 61)	43.3 ± 3.6 (30, 58)	0.7
Race (% Caucasian/% African American)	50.0/37.5	75.0/12.5	0.3
Sex (% female)	37.5	50	0.6
BMI (mg/kg^2^)	29.7 ± 2.4 (19.6, 41.7)	31.0 ± 1.4 (25.6, 35.5)	0.6
Body fat %	34.4 ± 2.3 (27.3, 43.8)	34.6 ± 2.0 (27.0, 42.9)	0.9
Fasting Glucose (mg/dL)	91.3 ± 3.4 (75.3, 102)	93.5 ± 3.1 (84, 109)	0.8
Fasting Insulin (uU/mL)	25.7 ± 12.3 (5.4, 103.3)	12.9 ± 1.7 (7.5, 22.9)	0.4
HOMA-IR	6.5 ± 3.3 (1.2, 27.9)	2.9 ± 0.4 (1.5, 5.1)	0.5
Glucose AUC	15,850.4 ± 560.0 (13,884.8, 17,905.5)	17,088 ± 1336.1 (11,187.0, 21,712.5)	0.4
Insulin AUC	8505.8 ± 2386.3 (2863.5, 23,309.0)	5612.2 ± 742.5 (2597.1, 8699.6)	0.4
Duration of Current Antipsychotic or Mood Stabilizer Therapy (months) *	94.1 ± 28.8 (3, 228)	58.6 ± 21.6 (6, 180)	0.3
Years Since First Psychopharmacologic Treatment	23.1 ± 3.9 (7, 37)	19.1 ± 3.2 (6, 33)	0.4

**Table 2:** The 40 proteins with significant differences between AAP and mood stabilizer groups. Data are given as fold changes (mean ± s.e.). Cutoff for significance was set at *p* < 0.01 or if a protein was not detected in one group but detected in one group and present in at least half of samples of the other group. The mean of the normalized PA for each protein in the AAP biopsy samples was set to 1.00, and the fold changes were relative to AAP. ND; not detected. ^#^, only detected in a minimum of 4 out of the 8 AAP muscle samples or 4 out of the 8 MS muscle samples.

Gene Name	Protein Name	AAP	MS	*p*-Value
ADSSL1	Adenylosuccinate synthetase isozyme 1	1.00 ± 0.07	0.61 ± 0.05	0.0004
ALDH9A1	4-trimethylaminobutyraldehyde dehydrogenase	1.00 ± 0.18	3.54 ± 0.34	0.0001
ANXA1	Annexin A1	1.00 ± 0.10	0.47 ± 0.09	0.0012
ANXA11	Annexin A11	1.00 ± 0.15	0.33 ± 0.15	0.0073
ANXA5	Annexin A5	1.00 ± 0.10	0.62 ± 0.08	0.0091
ATP5D	ATP synthase subunit delta, mitochondrial	1.00 ± 0.10	2.02 ± 0.30	0.0098
BTBD10	BTB/POZ domain-containing protein 10	1.00 ± 0.24	1.87 ± 0.06	0.0084
C1QBP	Complement component 1 Q subcomponent-binding protein, mitochondrial	1.00 ± 0.14	0.50 ± 0.06	0.0038
CAPNS1	Calpain small subunit 1	1.00 ± 0.13	1.64 ± 0.15	0.0042
CKAP4	Cytoskeleton-associated protein 4	ND	1.00 ± 0.15	<0.01 ^#^
COL6A1	Collagen alpha-1(VI) chain	ND	1.00 ± 0.25	<0.01 ^#^
COL6A3	Collagen alpha-3(VI) chain	1.00 ± 0.25	ND	<0.01 ^#^
DDX1	ATP-dependent RNA helicase DDX1	1.00 ± 0.09	0.57 ± 0.06	0.0032
ECHS1	Enoyl-CoA hydratase, mitochondrial	1.00 ± 0.23	0.36 ± 0.05	0.0009
EEF2	Elongation factor 2	1.00 ± 0.11	0.63 ± 0.06	0.0088
FABP3	Fatty acid-binding protein	1.00 ± 0.14	1.71 ± 0.23	0.0088
FERMT2	Fermitin family homolog 2	1.00 ± 0.16	ND	<0.01 ^#^
FHL1	Four and a half LIM domains protein 1	1.00 ± 0.08	1.66 ± 0.14	0.0012
GDI1	Rab GDP dissociation inhibitor alpha	1.00 ± 0.05	0.42 ± 0.03	0.0017
HNRNPDL	Heterogeneous nuclear ribonucleoprotein D-like	ND	1.00 ± 0.24	<0.01 ^#^
HSPA6/7	Heat shock 70 kDa protein 6/7	1.00 ± 0.08	1.66 ± 0.18	0.0034
KPNB1	Importin subunit beta-1	1.00 ± 0.17	0.46 ± 0.06	0.0052
LMNA	Lamin-A/C	1.00 ± 0.07	1.88 ± 0.27	0.0077
MTHFD1	Methylenetetrahydrofolate dehydrogenase	1.00 ± 0.13	0.32 ± 0.04	0.0003
MYH3	Myosin-3	1.00 ± 0.31	5.41 ± 1.07	0.0017
NME1/2	Nucleoside diphosphate kinase A/B	1.00 ± 0.21	2.81 ± 0.52	0.0035
PARK7	Protein deglycase DJ-1	1.00 ± 0.08	0.61 ± 0.09	0.0045
PDIA6	Protein disulfide-isomerase A6	1.00 ± 0.10	0.53 ± 0.08	0.0050
PLCL1	Phosphoinositide phospholipase C	1.00 ± 0.08	1.59 ± 0.20	0.0074
PLIN4	Perilipin-4	1.00 ± 0.10	2.50 ± 0.46	0.0016
PRDX1	Peroxiredoxin-1	1.00 ± 0.13	1.57 ± 0.14	0.0099
PRDX2	Peroxiredoxin-2	1.00 ± 0.09	1.59 ± 0.12	0.0020
RPL13	60S ribosomal protein L13	ND	1.00 ± 0.13	<0.01 ^#^
RPN2	Dolichyl-diphosphooligosaccharide--protein glycosyltransferase subunit 2	1.00 ± 0.27	0.10 ± 0.02	0.0075
RPSA	40S ribosomal protein SA	1.00 ± 0.09	0.49 ± 0.04	0.0001
SRL	Sarcalumenin	1.00 ± 0.09	0.60 ± 0.05	0.0025
YWHAG	14-3-3 protein gamma	1.00 ± 0.15	0.46 ± 0.09	0.0058
YWHAH	14-3-3 protein eta	1.00 ± 0.15	0.43 ± 0.07	0.0031
YWHAQ	14-3-3 protein theta	1.00 ± 0.18	0.41 ± 0.04	0.0044
YWHAZ	14-3-3 protein zeta/delta	1.00 ± 0.19	0.40 ± 0.08	0.0030

**Table 3:** Enriched pathways based on the 40 proteins with a significant difference between AAP and mood stabilizers using Ingenuity Pathway Analysis.

Ingenuity Canonical Pathways	FDR *q*-Value	Proteins Assigned to a Pathway
p70S6K Signaling	0.000028	YWHAQ, YWHAG, YWHAH, EEF2, YWHAZ, PLCL1
Cell Cycle: G2/M DNA Damage Checkpoint Regulation	0.0001	YWHAQ, YWHAG, YWHAH, YWHAZ
14-3-3-mediated Signaling	0.0002	YWHAQ, YWHAG, YWHAH, YWHAZ, PLCL1
ERK5 Signaling	0.0003	YWHAQ, YWHAG, YWHAH, YWHAZ
Myc Mediated Apoptosis Signaling	0.0003	YWHAQ, YWHAG, YWHAH, YWHAZ
HIPPO signaling	0.0003	YWHAQ, YWHAG, YWHAH, YWHAZ
IGF-1 Signaling	0.001	YWHAQ, YWHAG, YWHAH, YWHAZ
PI3K/AKT Signaling	0.001	YWHAQ, YWHAG, YWHAH, YWHAZ
ERK/MAPK Signaling	0.007	YWHAQ, YWHAG, YWHAH, YWHAZ
Protein Kinase A Signaling	0.008	YWHAQ, YWHAG, YWHAH, YWHAZ, PLCL1
L-carnitine Biosynthesis	0.05	ALDH9A1
Diphthamide Biosynthesis	0.05	EEF2
Tetrahydrofolate Salvage from 5,10-methenyltetrahydrofolate	0.07	MTHFD1
Folate Polyglutamylation	0.07	MTHFD1
Apoptosis Signaling	0.1	CAPNS1, LMNA
Histidine Degradation III	0.1	MTHFD1
Folate Transformations I	0.1	MTHFD1
Calcium Transport I	0.1	ANXA5
Purine Nucleotides De Novo Biosynthesis II	0.1	ADSSL1

## Data Availability

Data is available upon request from authors.
